# The Inflammatory Continuum of Traumatic Brain Injury and Alzheimer’s Disease

**DOI:** 10.3389/fimmu.2018.00672

**Published:** 2018-04-09

**Authors:** Olga N. Kokiko-Cochran, Jonathan P. Godbout

**Affiliations:** Department of Neuroscience, Institute for Behavioral Medicine Research, The Ohio State University Wexner Medical Center, Columbus, OH, United States

**Keywords:** traumatic brain injury, Alzheimer’s disease, neuroinflammation, microglia, monocyte, macrophage

## Abstract

The post-injury inflammatory response is a key mediator in long-term recovery from traumatic brain injury (TBI). Moreover, the immune response to TBI, mediated by microglia and macrophages, is influenced by existing brain pathology and by secondary immune challenges. For example, recent evidence shows that the presence of beta-amyloid and phosphorylated tau protein, two hallmark features of AD that increase during normal aging, substantially alter the macrophage response to TBI. Additional data demonstrate that post-injury microglia are “primed” and become hyper-reactive following a subsequent acute immune challenge thereby worsening recovery. These alterations may increase the incidence of neuropsychiatric complications after TBI and may also increase the frequency of neurodegenerative pathology. Therefore, the purpose of this review is to summarize experimental studies examining the relationship between TBI and development of AD-like pathology with an emphasis on the acute and chronic microglial and macrophage response following injury. Furthermore, studies will be highlighted that examine the degree to which beta-amyloid and tau accumulation as well as pre- and post-injury immune stressors influence outcome after TBI. Collectively, the studies described in this review suggest that the brain’s immune response to injury is a key mediator in recovery, and if compromised by previous, coincident, or subsequent immune stressors, post-injury pathology and behavioral recovery will be altered.

## Introduction to Traumatic Brain Injury (TBI) and Alzheimer’s Disease (AD)

Traumatic brain injury is a significant health concern affecting millions of individuals worldwide. Within the United States (U.S.), the Centers for Disease Control and Prevention report that 1.7 million individuals sustain a TBI annually, and 5.3 million individuals live with TBI-related disabilities (1). Similar structured reporting is limited from the rest of the world. Nonetheless, systematic reviews indicate that more than 7.7 million individuals live with TBI-related disabilities in the European Union (2). Subsequent reviews indicate that increased motor vehicle use is associated with a rising incidence of TBI globally (2–4). A significant concern is that standardized reporting and categorization in epidemiological studies around the world is absent. Consequently, TBI has been described as a “silent epidemic” for multiple reasons. First, epidemiological reports likely reflect an underestimation of incidence, particularly for milder forms of brain injury. Second, without an accurate incidence rate, it is impossible to identify the true public health and economic consequence of brain injury, including caregiver burden. Third, survivors of mild to moderate brain injury often display delayed and task-specific impairments making chronic, time-dependent reporting essential in documenting long-term effects of TBI. Finally, many post-injury problems are not visible, including cognitive and emotional impairment. Together, these points emphasize the many challenges that we face in attempting to improve recovery following TBI.

Age is closely associated with the incidence of TBI and likely plays a critical role in mediating response to and recovery from brain injury. For example, in the U.S. children aged 0–4 years, adolescents aged 15–19 years, and adults aged 65 years and older are among the most likely to sustain a TBI. Post-injury hospitalization and death are most common in adults aged 75 years and older, suggesting that age at the time of injury and aging after injury are important mediators of long-term recovery. Although a TBI occurs in a matter of milliseconds, the biological consequences of a brain injury may last a lifetime. Indeed, TBI is recognized as an environmental risk factor for many neurodegenerative diseases such as AD, Parkinson’s disease (PD), and chronic traumatic encephalopathy (CTE). The molecular mechanisms that link TBI to development of neurodegenerative disease remain underexplored and few studies account for age-specific pathological response to and recovery from brain injury.

Alzheimer’s disease is a neurodegenerative disease that progresses from mild cognitive impairment to severe dementia over time (5). The disease is characterized by key neuropathological features, including extracellular accumulation of beta-amyloid (Aβ) protein in senile plaques (6) and intracellular aggregation of microtubule-associated protein tau (MAPT, tau) in neurofibrillary tangles (NFTs) (7). Importantly, both amyloid and neurofibrillary changes begin during preclinical AD when cognitive deficits are not apparent (8). In typical cases of AD, Aβ spreads from the frontal and temporal lobes to the hippocampus and limbic system. NFTs spread from the medial temporal lobes and hippocampus to the neocortex (9). Several lines of evidence point to a relationship between single-incident TBI and AD (10, 11). First, numerous population-based studies demonstrate that head injury during adulthood increases the risk of AD later in life (12–19) and reduces the time to onset of AD (20). Second, many animal studies show increased production and accumulation of amyloid precursor protein (APP), Aβ, and pathological tau following TBI (16, 21–28). Third, accumulation of APP and extracellular deposition of the 40- to 42-amino acid Aβ peptide in senile plaques has been identified in human brain tissue soon after severe TBI (29, 30). Fourth, a comprehensive immunohistochemical study by Ikonomovic and colleagues identifies increased neuronal APP and diffuse Aβ deposits along with diffuse tau immunostaining in neuronal cell somata and axons, as well as glial cells, in resected temporal cortical brain tissue after TBI (31). Intracellular aggregation of MAPT in NFTs was only present in a subset of older subjects (31). These and other studies indicate that there is a biological link between TBI and AD pathology, but the exact molecular pathways underlying this relationship are poorly understood and post-injury mechanisms that facilitate Aβ and tau pathology remain under investigation. This review will consider post-injury neuroinflammation as a malleable response that is closely associated with development of AD-like pathology, thereby supporting a relationship between TBI, neuroinflammation, and development of AD.

A longstanding concern with many clinical studies is reliance on self-report and use of diagnostic verbiage in medical records to identify a correlation between TBI and post-injury development of AD (32). Also, several clinical studies report that individuals with genetic predisposition to developing AD (ApoE4 risk alleles) display altered outcome after TBI making the distinction between environmental and genetic risk factors for post-injury recovery unclear (33, 34). Despite preclinical studies providing evidence for successful pharmacologic intervention, more than 30 phase-III clinical trials have failed to improve secondary injury outcome measures after TBI (35–37). Finally, several experimental studies have failed to show that TBI induces or worsens AD-related pathology (38, 39) with some even reporting a reduction in post-injury Aβ accumulation in transgenic mice (40, 41). Collectively, these results highlight the complex nature of TBI and emphasize the need to clearly define post-injury mediating factors that could be contributing to variability in experimental and clinical studies.

### Experimental Models of TBI

To date, no effective interventions are available to improve recovery following TBI (42–44). Thus, experimental models are, therefore, essential in better understanding post-injury pathology and identifying effective therapeutic treatments. This strategy presents additional challenges as each animal model reflects a specific type of TBI and does not fully recapitulate primary and secondary damage evident in human TBI (45) resulting in restricted translation (46). Nonetheless, experimental models represent a critical tool in defining precise mechanisms of primary and secondary damage following TBI, particularly when the data generated are evaluated within the context of the injury model used.

A variety of TBI models are used in experimental studies. While no single model recapitulates all aspects of human TBI, the neuroinflammatory response to injury occurs in a temporally distinct manner. Several excellent reviews are already available that describe contemporary experimental models of injury as well as the inflammatory response to injury [see reviews in Ref. (47–49)]. Here, we will provide a brief description of the models described in this review. Experimental models of TBI have historically been referred to as focal or diffuse, but increasing evidence indicates that even focal brain injuries cause diffuse damage that is not restricted to the site of injury. In addition, concussive, repetitive, and blast-related TBI are often defined as diffuse injuries; however, collectively referring to them as “diffuse” undervalues the variability of the primary insult. Thus, describing the key neuropathological features is a more appropriate strategy for distinguishing experimental models from one another (49). Focal contusion models include controlled cortical impact (CCI) (50), fluid percussion injury (FPI) (51, 52), weight drop (53, 54), penetrating ballistic-brain injury (PBBI) (55). As implied, an external force (impactor tip, fluid, weight, and inflatable probe, respectively) is used to induce TBI and can be manipulated to produce a mild, moderate, or severe brain injury (as defined by post-injury pathology). Predominant pathological features include a focal contusion, blood–brain barrier (BBB) disruption, edema, in addition to neuronal and axonal damage. There is a widespread inflammatory response, including microglial and astrocytic activation, infiltration or peripheral cells, and increased production and release of inflammatory molecules which are reported up to 1 year post-injury (56). Recently, modified versions of the CCI without craniectomy have been employed to study single and repetitive forms of TBI (57, 58). In studies examining single or multiple mild severity TBI, skull fracture and cortical contusion are absent but neuroinflammation and behavioral impairment persist with increasing number of injuries (57). Studies discussed in this review include 2–30 injuries, with 30 injuries considered a highly repetitive model of TBI (59). In studies examining severe TBI, skull fracture and cortical contusion are induced *via* electrical weight drop on the exposed skull [closed head injury (CHI) model (60)]. Cryogenic brain injury is created when a cotton tip applicator dipped in liquid nitrogen is pressed on top of the skull. This type of injury does not directly induce cortical contusion but facilities an inflammatory response (61). Finally, a chronic hippocampal lesion model of brain injury will be discussed to highlight the effects of microglial elimination in post-injury outcome. The tetracycline-inducible promoter system is used to regulate neuronal expression of diphtheria toxin A-chain in this transgenic mouse model of injury. As a result, forebrain neurons expressing calcium-calmodulin kinas II α (CaMKIIα) are ablated resulting in neuronal loss, inflammation, and behavioral impairment (62).

In summary, these models of experimental TBI induce a temporal inflammatory response that is consistent with what is observed in human head injury, and increased injury severity is positively correlated with BBB disruption and infiltration of peripheral cells (63). Inflammatory cytokines and chemokines are immediately release after moderate TBI and peak within hours post-injury. Consequently, peripheral cells, such as neutrophils, monocytes, T-cells, and dendritic cells, enter the brain within days post-injury. Similarly, microglia and astrocyte reactivity increases within days post-injury, but altered and reactive morphology diminishes by 10–14 days post-injury. Chronic microglial and astrocytic reactivity, as defined by altered morphology, persists in sub-cortical brain regions months to years post-injury. Experimental TBI, regardless of model, consistently induces an inflammatory response including microglial/macrophage reactivity. This conserved response is, therefore, viewed as a critical mediator of post-injury outcome.

### Inflammation as a Mediator of Post-Injury Outcome

Primary damage occurs as a result of the physical and mechanical forces of brain injury and includes brain contusion, hemorrhage, hematoma, and axonal injury (3). Secondary damage develops hours and days after the primary damage, but is not necessarily dependent on the primary injury itself. In other words, secondary injury cascades can act concurrently and synergize to influence outcome (3). Secondary damage includes excitotoxicity (64), oxidative stress (65), and widespread neuroinflammation (63). Successful management of post-injury recovery is dependent on effectively stratifying these variables to determine which are predictive in outcome. We propose that the brain’s immune response to injury is a key mediator in recovery, and if compromised by *previous, coincident*, or *subsequent* immune stressors, progressive impairments will be evident.

Inflammation following TBI is a complex and dynamic response of both the central and peripheral nervous systems, which is influenced by age, sex, injury location and severity, secondary injury cascades, and genetics (63). Multiple other reviews eloquently describe this inflammatory process and provide insight into cell types and molecular pathways involved in the response (48, 63, 66, 67). Because inflammation occurs after all brain injuries, some propose that immune modulation is an integral component to identifying effective and clinically relevant therapeutic interventions (68). It is necessary to acknowledge that post-injury inflammation poses both beneficial and detrimental consequences that need to be balanced. A detailed understanding of mechanisms driving immune activation after TBI is, therefore, of utmost importance (67). In this review, we would like to extend the conversation to include appreciation of the inflammatory continuum that occurs over a lifetime. TBI is not an isolated event within the inflammatory milieu. Accumulating data indicate that pre- and post-injury immune challenges may influence the microglial and macrophage response to brain injury and influence post-injury pathology and behavioral recovery.

### Relevance of Microglia and Macrophages in TBI

This review will focus on the role of brain-resident microglia and infiltrating peripheral monocytes. In normal physiological conditions, the BBB prevents entry of peripheral monocytes into the brain parenchyma. Disruption and dysfunction of the BBB after TBI facilities monocyte infiltration though. When in the reactive macrophage state, it is difficult to distinguish microglia and peripheral monocytes. For example, after TBI, microglia and monocyte-derived macrophages adopt a similar morphology, upregulate similar inflammatory surface markers, and increase production of similar inflammatory cytokines. Indeed, many prevalent immunohistochemical markers do not identify whether or not a reactive macrophage is of microglial or monocytic origin. Given the distinct genetic and transcriptomic profile of microglia and macrophages identified *via* high throughput sequencing technology (69–71), the two cell types likely maintain different roles in the injured central nervous system (CNS) (72–74).

Targeted genetic deletion of key chemokine receptors, CCR2 and CX3CR1, has emerged as a useful tool to characterize the role of microglia and macrophages following TBI (48). The surface glycoprotein Ly6C can be used in combination with CCR2 and CX3CR1 to identify two distinct populations of monocytes in peripheral blood, Ly6C^high^/CX3CR1^low^/CCR2^+^ and Ly6C^low^/CX3CR1^high^/CCR2^−^. The former is the inflammatory subset of monocytes that differentiate into inflammatory macrophages in response to post-injury inflammation. The latter is the patrolling subset of monocytes that survey the vasculature and resolve inflammation. CCR2 is required for monocytes to enter the CNS and, therefore, all infiltrating monocytes are CCR2^+^; however, downregulation of CCR2 following CNS entry has been reported. Genetic deletion of CC ligand-2 (CCL2), the cognate ligand for CCR2, attenuates lesion volume, reduces macrophage recruitment and astrogliosis, and improves functional outcome compared to controls after CHI (60). Similarly, post-injury macrophage recruitment substantially decreases in *Ccr2^−/−^* mice following CCI TBI and correlates with improved behavioral outcome (75). Separate groups have shown that CCX872 and RS504393, two selective CCR2 antagonists, reduce post-injury macrophage recruitment and improve functional recovery after CCI and weight drop TBI (76, 77). Together these studies indicate that interruption of CCL2/CCR2 signaling offers therapeutic potential to improve outcome following TBI and lends support to the notion that a persistent post-injury macrophage response is detrimental to outcome. Interruption of CCR2 signaling through the use of *Ccr2^RFP^*^/^*^RFP^* mice reduces post-injury monocytic infiltration and axonal pathology but enhances cortical and hippocampal MAPT mislocalization and hyperphosphorylation soon after lateral fluid percussion TBI suggesting that monocyte sub-populations may differentially influence outcome (78). Without detailed follow-up studies, the roles of monocyte sub-populations in mediating outcome from TBI remain unknown. Collectively, these studies indicate that interruption of post-injury monocytic infiltration has both beneficial and detrimental consequences depending on the outcome measures evaluated.

The microglial response to TBI has been explored *via* genetic manipulation of CX3CR1. For example, fractalkine (CX3CL1) and its cognate receptor CX3CR1 represent a unique one-to-one ligand–receptor pair. In the CNS, CX3CL1 is highly expressed in neurons and CX3CR1 is expressed by microglia from their first entry into the neuroepithelial parenchyma around E10 throughout adulthood (79). Microglia remain uniformly CX3CR1^+^ and do not express CCR2 or downregulate CX3CR1, even during severe neuroinflammation evident after TBI. After a single CCI TBI, *Cx3cr1^−/−^* mice have improved motor recovery and decreased neuronal loss through 15 days post-injury (DPI). By 30 DPI, however, these *Cx3cr1^−/−^* mice have worse cognitive dysfunction and neuronal loss compared to wild-type controls. These changes are directly associated with an altered and time-dependent inflammatory profile in microglia (80). Subsequent work by a separate group confirms these results and demonstrates that CX3CR1 deficiency results in early protection but chronic worsening of CCI TBI-induced deficits due in part to a decrease in anti-inflammatory cytokines on CD11b^+^ sorted cells at 28 DPI (81). Together, these studies emphasize the temporal inflammatory response to a single brain injury and confirm that alteration of this response can influence outcome. Moreover, acute transient interruption of the microglial response to TBI is beneficial to outcome.

Use of *CX3CR1^GFP/+^CCR2^RFP/+^* reporter mice provides insight into the role of microglia and macrophages following TBI (76); however, it is still unclear if myeloid cells associated with chronic injury lesions are CX3CR1^+^ microglia or a mixture of CX3CR1^+^ microglia and CX3CR1^+^ peripheral macrophages that downregulated CCR2. Future studies geared to address the therapeutic potential of targeting specific sub-populations of reactive macrophages may hold great translational significance. Because the cell-specific role of microglia and blood-derived macrophages in post-injury recovery remains limited, they will be collectively referred to as macrophages within this review.

## Post-Injury Neuroinflammation and Aging

Accumulating evidence implicates the post-injury inflammatory response as a key mediator in long-term recovery from TBI. Many biological pathways are disrupted by experimental TBI resulting in progressive neurodegeneration including atrophy, neuronal loss, and axonal degeneration which are often associated with neuroinflammation including macrophage reactivity (82–85). These findings are consistent with human studies that report increased mRNA expression of microglial markers OX-6 and CD68 at 1 year post-injury (11) and imaging studies showing increased binding of PK-[11C](R)PK11195 ligand, expressed by activated microglia, between 11 months and 17 years post-injury (86). Macrophage-mediated neuroinflammation is also a prominent feature of many age-related neurodegenerative diseases including AD (87). For example, myeloid cells are instrumental in maintaining CNS homeostasis; however, aging significantly alters their properties (88). Consequently, age-related immune changes and those that occur during AD share many similarities and the distinction between the two processes remains unclear (89). Determining the extent to which age-related impairments in myeloid functioning facilitates accumulation of Aβ or if accumulation of Aβ impairs myeloid functioning is critical in identifying which immune pathways should be targeted (90). Moreover, inflammation is a malleable response to TBI that changes with aging which suggests that it could be critical in mediating post-injury outcome.

Age-related changes in the function of microglia and macrophages may influence outcome after TBI. For example, phagocytosis and chemotaxis diminish in both microglia and macrophages during aging. While the age-related pro-inflammatory response to immune challenge is decreased in macrophages (91), aged microglia displayed an exaggerated pro-inflammatory response referred to as “microglial priming” first described in a model of prion disease (92). A primed microglia profile includes (1) increased basal expression of inflammatory markers and mediators, (2) decreased activation threshold to express and release pro-inflammatory molecules, and (3) exaggerated inflammatory response to immune challenge (93). The cause of microglial priming is unclear and likely results from multiple factors, including but not limited to (1) a loss of inhibitory ligand–receptor communication with aging neurons (94), (2) interactions with age-related misfolded proteins such as Aβ which promote pro-inflammatory cytokine production (95), (3) age-related exposure to increasing transforming growth factor-β which could compromise microglial transitioning from a pro- to anti-inflammatory phenotype (96), (4) age-related alterations in production of IL-4 and CCL11 in the choroid plexus (97–99), and (5) unique microenvironment effects in white and gray matter. For example, previous studies demonstrate that gray matter injury elicits an enhanced macrophage response in older rodents compared to younger rodents (100, 101); however, white matter demyelination injury provokes a reduced macrophage response in older rodents compared to younger controls (102). Collectively, these data indicate that aging before and after TBI could significantly influence outcome.

Taken together, these findings indicate that the immune response to and recovery from TBI is not absolute and very much influenced by multiple factors. Existing brain pathology and secondary immune challenges may be critical in shaping post-injury disease pathogenesis. Indeed, macrophage-mediated inflammation across the continuum of aging should be considered in the context of TBI, particularly when studying outcome related to development of neurodegenerative disease. Therefore, the primary purpose of this review is to summarize studies examining the relationship between single-incident TBI and development of AD-like pathology with an emphasis on the acute and chronic microglia and macrophage response following injury. Repetitive TBI will be considered as a repeated immune stressor and discussed only briefly. Furthermore, studies will be highlighted that examine the degree to which pathological protein accumulation and peripheral immune stressors influence outcome after TBI.

### TBI, Inflammation, and AD

Chronic inflammation is a potential common denominator in both TBI and AD. TBI induces a widespread neuroinflammatory response that can promote recovery if controlled for a defined time period. Excessive or chronic neuroinflammation is linked to progressive changes, including atrophy, neuronal loss, and axonal degeneration (84, 103–105). Post-injury neuroinflammation is characterized by activation of brain-resident microglia, infiltration of peripheral immune cells, astrogliosis, and increased synthesis and release of pro- and anti-inflammatory molecules which can persist for months to years after the initial insult (106, 107). There is a persuasive body of evidence showing a significant inflammatory component in AD as well. First, microglia, monocytes, and astrocytes as well as inflammatory cytokines and chemokines are elevated in the AD brain (108). Second, retrospective studies demonstrate that sustained NSAID treatment during mid-life significantly decreases the risk of AD (109, 110). Considering the failure of prospective studies with NSAID treatment (111), the beneficial effects of NSAIDs is presumed to be related to pre-morbid function. Third, recent genetic studies implicate inflammatory genes and pathways (*CD33, TREM2, HLA-DRB5-*DRB1) in late-onset disease pathology (112–115). Fourth, alterations in inflammatory cells and molecules are reported in multiple different mouse models of AD. Finally, accumulating evidence shows that microglia and monocytes play distinct roles in AD pathogenesis (116–119), thus implicating both the central and peripheral immune response in long-term outcome. Collectively, these results suggest that chronic post-injury neuroinflammation may be sufficient to induce or facilitate AD-related pathology.

## TBI and Amyloid-Related Pathology

Rodent models have been a valuable resource in studying the relationship between TBI and AD-like pathology [see reviews (10, 120)]; however, most of the early studies focused on accumulation and production of Aβ. Many types of CNS injury, including TBI, induce the expression of APP. For example, APP expression increases in striatal and hippocampal axons along with cortical and thalamic neurons within the first 24 h after experimental impact and fluid percussion TBI (121–123) which has been replicated in multiple follow-up studies using CCI as well as midline and lateral FPI (124–126). Traumatic axonal injury (TAI) is an additional source of accumulating APP (127, 128). For example, APP accumulates in traumatized axons after all severities of TBI and has been detected many months post-injury (129–131).

Amyloid precursor protein accumulation does not result in Aβ deposition in many experimental studies though. Although Aβ deposition is apparent following rotational acceleration TBI in pigs (132, 133) and rabbits (134), a majority of rodent studies fail to show this association in non-transgenic animals using CCI, FPI, and weight drop models (121, 122, 124, 125, 135). Consequently, the validity of TBI-AD experiments in non-transgenic rodents is unclear. Many factors likely contribute to the lack of Aβ deposition in these studies. For example, multiple reports indicate that there are endogenous differences in rodent and human APP (136), which could significantly alter the production on Aβ after TBI. Injury severity may also be a critical mediator in outcome. Clinical studies indicate that Aβ accumulates within hours after severe TBI and is spread throughout the cerebral cortex compared to age-matched controls (29, 31). Indeed, the complex neuroanatomy and neurophysiology of the human brain, such as cortical folding, substantial white matter, and specific pathophysiology compared to the rodent brain, may facilitate distinct post-injury neuropathology (137). Finally, location and timing of injury may mediate Aβ pathology. For example, Aβ accumulation is observed in patients with *dementia pugilistica* which reflects traumatic injury as a result of repetitive brain insults. Thus, Aβ may have a specific temporal profile in single and repetitive models of TBI.

The availability of transgenic and knock-in mouse models of AD expressing wild-type or mutant human APP provided an additional avenue of study to determine the relationship between TBI and amyloid-related pathology. One of the earliest mouse models of AD utilized a platelet-derived growth factor-β promoter to overexpress mutant human APP. These PDAPP transgenic mice display age-related cognitive impairment, synaptic dysfunction, Aβ accumulation, and tau phosphorylation. Although CCI TBI induced a surge of plaque pathology in PDAPP mice soon after injury at 6 months of age, a substantial reduction in cortical and hippocampal plaque load was detected chronically (28, 40). Follow-up experiments revealed that CCI TBI in aged PDAPP mice caused a regression of established Aβ deposits (41). In both sets of experiments, the reduction in Aβ pathology was accompanied by increased neuronal death and memory impairment, ultimately bringing into question the neurotoxic properties of Aβ alone.

Monomeric Aβ aggregates to form oligomers, protofibrils, and fibrils that accumulate in the characteristic Aβ plaque of AD. Thus, the production of Aβ is a complex process and accumulating evidence indicates that soluble Aβ oligomers, not Aβ plaques alone, are the disease-causing species that induce substantial neurotoxicity including synaptic dysfunction and behavioral impairment [see review in Ref. (138)]. Surprisingly, the role of soluble Aβ oligomers in post-injury pathology has received limited attention. While several clinical studies report that higher cerebral spinal fluid (CSF) levels of Aβ42 predict improved neurological recovery following severe TBI (139, 140), higher levels of CSF Aβ oligomers predict poor neurological recovery (141). A single experimental study examined the accumulation of soluble, insoluble, and oligomeric Aβ following TBI in the 3xTg mouse model of AD which harbors (overexpressed) transgenes carrying genetic mutations that promote Aβ and tau pathology. While CCI TBI increased soluble and insoluble cortical Aβ40 and Aβ42 within 24 h after injury, both soluble and insoluble Aβ returned to sham levels by 7 DPI (23). Although these studies indicate that TBI induces an acute increase in oligomeric Aβ, the long-term consequences of this increase and the effect on specific cell types or brain region pathology remains unknown.

Based on the abovementioned results, one might suggest that the validity of TBI-AD experiments in APP-transgenic rodents is unclear as well. Indeed, many of these models express mutant APP at higher levels than endogenous APP and maintain genetic risk variants that cause familial AD which is fairly uncommon. The co-occurrence of TBI and APP mutation in the clinical setting is rare (29) thereby restricting the results of many of these studies. Recent findings shed light on the discrepancies between experimental TBI-AD studies and emphasize the potential role of non-neuronal cells in mediating outcome. For example, TBI in the APP/PS1 knock-in mouse model of AD results in a delayed neuroinflammatory response compared to wild-type control mice subjected to CHI (16). While both brain-injured AD and wild-type mice had increased expression of inflammatory cytokines IL-1β, IL-6, and TNFα, peak elevations were delayed by 7 days in the AD mice but persisted in conjunction with astrocyte activation. A similar trend was observed in the chemokines CCL2, CCL3, CCL4, and CCL5. In addition, mRNA expression of CCR2, CD68, and MHC-II, characteristically expressed by macrophages, was delayed in APP/PS1 mice compared to wild-type controls following TBI. Treatment with MW151, a small-molecule inhibitor targeting pro-inflammatory cytokines in glia, attenuated the persistent increase in pro-inflammatory cytokine expression and improved cognitive recovery in APP/PS1 mice. Collectively, these results indicate that there is a direct relationship between neuroinflammation and functional recovery and emphasize the distinct temporal inflammatory response to TBI in APP/PS1 mice (16).

The immunomodulatory effects of accumulating Aβ were confirmed in another set of TBI experiments. A separate group of investigators examined the macrophage response to lateral FPI in the R1.40 mouse model of AD, which maintains genetic predisposition to developing Aβ deposits between 12 and 15 months of age *via* multiple copies of the mutant APP yeast artificial chromosome (38). TBI was administered to young, 2-month-old mice to determine if brain injury worsened or advanced the appearance of age-related AD-like pathology. The acute macrophage response to TBI, as measured by Iba1, CD45, F4/80, CD68, and Trem2 immunohistochemistry, was strikingly muted in R1.40 TBI mice compared to wild-type mice exposed to TBI. Flow cytometry revealed that reduced numbers of myeloid cells acquired a macrophage phenotype in R1.40 TBI mice, correlating with decreased inflammatory cytokine expression. At a chronic time point, several months after TBI, the macrophage response to injury subsided in wild-type mice; however, it was relatively unchanged in R1.40 mice. In addition, R1.40 mice displayed significant tissue loss between 3 and 120 DPI and task-specific cognitive deficits in transferring information from 1 day to the next at 120 DPI. Importantly, TBI did not advance the appearance of Aβ plaques in R1.40 mice. Together, these findings emphasize the potential neuromodulatory role of accumulating Aβ and demonstrate that the glial response to TBI is altered in the presence of Aβ and correlates with altered functional recovery (38).

The immunomodulatory role of Aβ has been manipulated in other experimental models. For example, a 2013 study revealed that peripheral administration of Aβ42 and Aβ40 attenuates paralysis and reduces neuroinflammation in multiple mouse models of experimental autoimmune encephalomyelitis (EAE) (142). Aβ specifically suppressed cytokine secretion in activated peripheral lymphocytes and reduced inflammatory foci within the CNS without promoting Aβ deposition in the brain. These results indicate that Aβ maintains both pathological and beneficial properties which are dependent on the type of CNS injury and the inflammatory context, namely lymphoid or brain tissue. Follow-up studies show that a potent hexapeptide core structure in amyloid is highly immunosuppressive and likely mediating these effects to some degree (143). A 2016 study demonstrates that Aβ is anti-microbial and protects against *Salmonella enterica* serotype Typhimurium (*S*. Typhimurium) infection in the 5XFAD transgenic mouse model of AD potentially *via* oligomerization. *S*. Typhimurium infection induced Aβ deposition in 1-month-old 5XFAD mice compared to control 5XFAD mice, which appeared to surround and entrap bacterial colonies (144). The idea of using Aβ as a therapeutic is directly contrary to Aβ strategies in AD, which aim to remove Aβ from the brain. Nonetheless, these studies highlight a physiological role for Aβ in innate immunity and emphasize the effect of Aβ on other cell types which directly influences disease pathogenesis and functional outcome.

What does this mean for experimental TBI-AD research? In fact, the role of Aβ in mediating response to and recovery from TBI is largely unknown and may contribute to the variability in experimental and clinical studies examining the relationship between the two pathologies. For example, many studies report the presence or absence of Aβ as a primary dependent variable of interest following TBI with little attention given to oligomers, protofibrils, and fibrils. Based on recent evidence, the presence of these low-molecular weight aggregates may substantially alter the neuroinflammatory environment and influence outcome following TBI. Given that Aβ alone is not predictive of AD and many older neuropsychologically healthy individuals display amyloid deposition (145, 146), age-related Aβ accumulation may play a critical role in the brain’s ability to respond to and recover from traumatic injury. Many techniques are available to identify cell-specific changes following TBI which could be incorporated into future experimental TBI studies. For example, generating AD mice with targeted deletion of CCR2 or CX3CR1 could provide information on the cell-specific response of microglia and monocytes to TBI in the presence of accumulating Aβ. Subsequent fluorescence-activated cell sorting (FACS) would allow investigators to identify the cell-specific inflammatory profile of microglia and monocytes in this context. In addition, laser capture microdissection of macrophages near and away from Aβ plaques could be useful in identifying the spatial influence of Aβ accumulation. Finally, consistent inclusion of non-transgenic control mice would provide investigators with an opportunity to determine if transgenes of interest influence the response to and recovery from TBI.

## TBI and Tau-Related Pathology

Tau is a scaffolding protein found in neurons and enriched in axons where it regulates microtubule assembly primarily *via* phosphorylation. Increased tau phosphorylation reduces microtubule affinity and supports neuronal plasticity and axonal transport at the synapse (147). Under pathological conditions, such as those occurring in AD, increased post-translational modification of tau facilitates aggregation and impaired clearance from the brain resulting in characteristic NFTs [see review in Ref. (148)]. TBI-induced axonal injury is proposed to be the first perturbation of tau resulting in dissociation from the microtubules. A robust and persistent neuroinflammatory response may then be sufficient to promote phosphorylation, aggregation, and subsequent neurodegeneration; key features of AD (149–151). For example, multiple experimental models of TBI enhance tau pathology that temporally co-exists with gliosis (21, 152, 153). In addition, activated microglia near the injury site release pro-inflammatory cytokines and chemokines that exacerbate tau pathologies (153–155). This is consistent with what is observed in many other tauopathies (156–159), including AD (160); reactive microglia correlate with tau lesions. Together, these studies indicate that chronic neuroinflammation could provoke tau pathology thereby worsening neuronal injury and long-term outcome. Controversy remains in this area though [see review in Ref. (161)], and some data suggest that senescent rather than reactive microglia drive tau pathology and neurodegeneration in AD (162–164). While the relationship between neuroinflammation and neurodegeneration remains complex, a breakdown in communication between microglia and neurons likely sets the stage for neuropathology.

Collectively, human studies show that post-injury tau pathology varies in response to severity, type, and number of brain injuries as well as the time point of post-injury analysis. For example, temporal excision soon after severe TBI reveals axonal and white matter tau phosphorylation in the absence of somatodendritic accumulation (31). Severe TBI resulting in death induces sporadic phosphorylated tau and tau-positive glia but no difference in NFT pathology compared to age-matched controls (165, 166). Together, these studies demonstrate that single TBI induces acute tau phosphorylation but not aggregation. Other studies show that a history of single-incident TBI increases amyloid and tau pathology, neuroinflammation, and white matter degeneration compared to age-matched controls many years after the initial injury (11, 167). Tau pathology, in particular, extended beyond the entorhinal cortex and hippocampus to the cingulate gyrus, superior frontal gyrus, and insular cortex, which was not observed in controls (167). The co-localization of tau pathology and neuroinflammation was not depicted in these studies. Tau pathology has been consistently reported after mild repetitive TBI resulting in CTE. Historic studies on CTE were in boxers, but recent evidence indicates that athletes in many impact-related sports have increased tau pathology followed repetitive mild TBI [see review in Ref. (168)]. Finally, TBI resulting from exposure to an explosive blast causes axonal injury, tau phosphorylation, persistent neuroinflammation, and neurodegeneration characteristic of CTE suggesting that common pathogenic mechanisms mediate outcome in military veterans and repetitively injured athletes (152, 169). These data indicate that tau phosphorylation is a conserved response to TBI regardless of primary insult, but progressive tau pathology occurs in response to repetitive or blast TBI.

Experimental studies indicate that post-injury tau pathology is variable and dependent on multiple factors in non-transgenic rodents. Overall, tau phosphorylation is commonly reported soon after single-incident CCI, weight-drop, FPI, and blast TBI (<7 DPI) (170–174); however, chronic worsening of tau pathology is rare. For example, single blast TBI induces tau phosphorylation in the cortex and hippocampus at 30 DPI (175), with separate groups also reporting persistent hippocampal pathology at 3 months post-injury (176). Also, cortical and hippocampal tau phosphorylation is reported 6 months after moderate CCI but not 6 or 12 months after mild CCI (58, 177). Tau phosphorylation is only part of a potentially pathological process. Following hyperphosphorylation, tau self-assembles, aggregates, and forms NFTs; however, tau oligomers may represent the most toxic and pathologically relevant aggregate. Indeed, oligomeric tau contributes to neurotoxicity by disrupting mitochondrial and synaptic function and strongly correlates with behavioral impairment (178). Recent studies show that fluid percussion TBI induces oligomeric tau in the cortex and hippocampus within 24 h post-injury where it remains elevated compared to shams 2 weeks post-injury (179). Post-injury oligomeric tau was isolated from TBI mice in follow-up studies and injected in the hippocampus of mice overexpressing human tau (hTau). Tau oligomers derived from brain injured mice subsequently caused cognitive dysfunction and the appearance of tau oligomers in hTau mice supporting the notion that tau oligomers are neurotoxic and contribute to tau spreading throughout the brain (180). Accumulating evidence indicates that neuron-to-neuron propagation of tau is a key feature of neurodegenerative tauopathies including AD [see review in Ref. (181)]. Together, these studies implicate soluble tau aggregates as mediators of pathological spreading throughout the brain termed “cistauosis.” Thus, abnormal processing of tau is not necessarily the primary mechanism of disease pathogenesis. Recent studies support this concept and indicate that both blast and impact TBI induce *cis* p-tau leading to axonal disruption, tau spreading, and neurodegeneration. Treatment with *cis* p-tau antibody consequently blocked pathological tau spreading and improved functional recovery (182). Collectively, these results indicate that tau alone possesses neurotoxic properties that mediate recovery following TBI.

Few experimental TBI studies have been performed in tau transgenic mice without concurrent amyloid pathology. Single-incident mild CHI TBI in aged hTau transgenic mice that express all six isoforms of hTau in absence of murine tau did not worsen tau phosphorylation or induce tau aggregation 3 weeks post-injury (153). A separate group of investigators examined the acute and chronic effects of moderate lateral FPI in a similar hTau mouse model, mouse tau knockout expressing wild-type human transgene, and found that the macrophage response to TBI was enhanced compared to control TBI and sham mice at 3 DPI with no influence on tau phosphorylation (183). This was confirmed with immunohistochemistry examining expression of CD45, F4/80, and CD68. By 120 DPI hTau TBI mice displayed increased tau pathology in the cortex and hippocampus and a persistent macrophage response that correlated with deficits in spatial search strategies to complete a memory task (183). Incorporation of flow cytometric techniques facilitated identification of four distinct macrophage populations at 120 DPI: (1) CD11b^low^/CD45^low^ microglia, (2) CD11b^high^/CD45^low^ microglia, (3) CD11b^+^/CD45^int^ microglia, and (4) Ly6C^+^/CD11b^+^/CD45^high^ macrophages. The CD11b^low^ microglia expressed the lowest levels of CD45, followed by the CD11b^high^ and the CD45^int^ groups characteristic of reactive microglia, while the peripheral macrophages were the highest expressers of CD45. A significant proportional reduction was identified in hTau TBI compared to wild-type TBI mice in all three microglial sub-populations at 120 DPI. No significant differences were observed in the proportion of CD11b^+^/CD45^high^ cells between brain- and sham-injured hTau and wild-type mice. Ly6C^low^ and Ly6C^high^ microglia were significantly reduced in the hTau TBI mice, but Ly6C^low^ macrophages persisted at significantly higher numbers compared to the hTau sham-injured group. The authors speculate that Ly6C^low^/CD11b^+^/CD45^high^ cells represent CX3CR1^+^ patrolling macrophages (184), and that Ly6C^+^/CD11b^+^/CD45^int^ microglia represent inflammatory CCR2^+^ monocyte-derived macrophages, differentiating in the CNS tissue environment. Without detailed cell-specific analysis of cytokine and chemokine expression, the true nature of these cell populations remains unclear. For the first time, these results show that a single TBI significantly changes the proportion of reactive microglia and macrophages within the brains of hTau mice compared to wild-type mice many months after TBI (183). These data indicate that the presence of wild-type hTau is sufficient to alter the macrophage response to single-incident TBI.

Collectively, these studies confirm the vulnerability of the brain to tau pathology following single-incident TBI. Indeed, both clinical and experimental studies consistently report tau phosphorylation soon after TBI; however, the presence or absence of tau phosphorylation alone is not sufficient to define tau pathology and may represent a transient effect of TBI. Furthermore, the role of tau oligomers is very limited in the context of TBI and represents an important avenue of study for future experiments. The prion-like properties associated with abnormal tau implicate the protein itself as an initiator of disease pathogenesis. As a result, the relationship between damaged neurons and other cells types remains unclear and the question remains, which cell is driving post-injury pathology? While recent experimental studies demonstrate a unique macrophage response to TBI that correlates with tau pathology and behavioral impairment, one is left wondering whether or not the abnormal tau caused the altered inflammatory response or the altered inflammatory response caused the abnormal tau? Certainly, use of tau knockout mice or CCR2 and CX3CR1 knock-in/knock-out mice could provide insight into these questions. In addition, the time course of pathology must be a priority. Defining age-related pathology requires aging as a primary variable of interest and the temporal course of disease pathology should not be undervalued.

## TBI and Combined Effect of Amyloid and Tau-Related Pathology

The combined effect of amyloid and tau pathology has gained recent attention over the last 10 years; however, results from non-transgenic rodent studies remain variable. The presence of Aβ and tau pathology appears to be dependent on the injury model used and the post-injury time point. Both fluid percussion and moderate CCI TBI induce Aβ and tau pathology at acute (3 and 7 DPI) and chronic (6 months post-injury) time points in rats (177, 185, 186), but other groups report no difference in Aβ or tau levels at 2 and 4 weeks post-injury (187). PBBI decreased full length APP at 3 and 7 DPI but increased beta-secretase C-terminal fragments of APP. Both Aβ40 and Aβ42 were increased at 7 DPI, but the authors explain that detection was difficult due to low expression. Similarly, full length tau decreased at 3 and 7 DPI but oligomeric tau was elevated at 4 h and 7 DPI (188). Out of these studies, only one reported that increased Aβ and tau pathology occurred in conjunction with neuronal loss and increased MHC-II immunoreactivity several months post-injury (177).

Given that Aβ and abnormal tau are hallmark features of AD, transgenic mice harboring mutations in both APP and MAPT are more often used to characterize the relationship between TBI and AD. Use of these models provides investigators with an opportunity to study the interaction of Aβ and tau pathology following TBI, but the clinical relevance of these models often comes into question. To date, no mutation in MAPT is causative in development of AD thereby restricting the translation of results. Nonetheless, accumulation of Aβ and tau pathology occurs as a result of normal aging [see review in Ref. (189)] and, therefore, the relevance of these abnormal proteins as mediators of response to and recovery from TBI remains applicable.

Variations of CCI have been used to examine the effects of TBI mouse models of AD with amyloid and tau mutations *via* overexpression of transgenes. For example, a series of studies examining moderate CCI in 3xTg-AD mice revealed a temporally and anatomically distinct increase in intra-axonal Aβ and tau phosphorylation between 24 h and 7 DPI (21, 25). Follow-up studies revealed that post-injury Aβ and tau pathology could be improved *via* inhibition of γ-secretase or c-Jun N-terminal kinase (JNK), respectively (21, 190). Interestingly, a recent report shows that post-injury JNK inhibition improves amyloid and tau pathology, neuroinflammation, BBB disruption, synaptic loss, and neurodegeneration in non-transgenic mice 7 DPI (191). Thus, the JNK pathway may be a relevant therapeutic target influencing multiple pathological processes.

In addition, the effect of *ApoE4* allele was examined in the 3xTg mice after TBI. *ApoE4* is a primary genetic risk factor for late-onset AD and has been associated with worsened outcome after TBI (192–196). While 3xTg-ApoE4 mice displayed increased post-injury APP accumulation compared to 3xTg mice with the *ApoE2* or *ApoE3* allele, TBI did not influence intra-axonal Aβ40 and Aβ42 or tau pathology 24 h post-injury. These results demonstrate that axonal injury may be a primary effect of *ApoE4* genotype following TBI but the interaction effect of *ApoE* and tau pathology remains unclear in this experimental model of TBI (197). Collectively, these data indicate that genetic predisposition to AD drives independent mechanisms that promote post-injury amyloid and tau pathology.

Other studies have examined tau pathology in mouse models of AD with genetic predisposition to developing only amyloid pathology *via* inclusion of mutant human transgenes. A recent study examining CCI in APP/PS1 mice revealed chronic region-specific changes in Aβ with no change in tau pathology 16 weeks post-injury (198). For example, Aβ plaques decreased in the perilesional area after TBI which correlated with increased expression of genes involved in Aβ clearance (198). Finally, CCI in Tg2576 mice, which overexpress mutant APP, increased Aβ, tau phosphorylation, and inflammatory cytokines IL-1β and TNF-α 3 DPI. Inhibition of GSK *via* treatment with the flavonoid luteolin attenuated this response (199), but the long-term and functional consequences of this intervention remain unknown.

Together, these studies indicate that choice of experimental TBI model and rodent model (rat or mouse, transgenic or non-transgenic) influence the temporal appearance of post-injury amyloid and tau pathology through independent mechanisms. Alternatively, a common mechanism may mediate post-injury amyloid and tau pathology in a temporally distinct manner which may vary between transgenic and non-transgenic rodent models. Finally, one could hypothesize that accumulating pathological proteins in transgenic rodents substantially mediates the brain’s ability to respond to injury by priming the inflammatory environment *before* TBI. Cell-specific inflammatory profiles of microglia and monocytes are not routinely performed in transgenic mice prior to TBI, therefore this effect remains unknown. In summary, the combined effect of amyloid and tau pathology following TBI is complex and likely dependent on multiple factors that are time- and injury severity-dependent. We propose that future studies look beyond accumulation of amyloid and tau as primary dependent variables of interest and consider interaction effects of inflammation, amyloid, and/or tau as mediating factors of post-injury outcome measures. Increasing evidence, as described in the following sections, clearly shows that pre- and post-injury immune stressors that elicit macrophage reactivity influence response to and recovery from TBI.

## Pre-Injury Peripheral Immune Challenge Improves Recovery Following TBI

Neuroprotective preconditioning occurs when a moderate primary stimulus protects the CNS from a secondary stimulus. The goal is to use a sub-threshold inflammatory stimulus to pre-condition a neuroprotective response to a secondary stimulus. For example, peripheral LPS treatment is neuroprotective against stroke, ischemia, and higher-dose LPS treatments (200). Similar effects have been reported in experimental models of TBI. For example, a single i.p. dose of LPS (0.1 mg/kg) 5 days before CCI reduced CD68 and increased IL-6 expression in TBI mice, which correlated with decreased contusion volume and improved behavioral recovery (201). Follow-up studies revealed that a single i.p. dose of LPS (0.1 and 0.5 mg/kg) 5 days before CCI delayed post-injury kindling epileptogenesis. In addition, pre-injury LPS treatment attenuated neuronal loss, IL-1β, and TNFα overexpression in the hippocampus (202). More recently, pre-injury treatment with LPS (1.0 mg/kg, single i.p. dose for 4 days) reduced neuronal death and lesion volume after lateral cryogenic brain injury (61). The authors conclude that microglial reactivity induced by 4 peripheral pre-injury LPS injections offers post-injury neuronal protection. Indeed, Chen and colleagues demonstrate that peripheral LPS treatment increases cortical expression of M2-related genes, such as *Ym1, Socs3, Il4ra, Ptprc, Cd163, Il1ra, Mrc1*, and *Arg1* (61). While the appearance of AD-related pathology was not examined in any of these studies, the data support a neuroprotective role of reactive microglia and indicate that pre-injury immune challenge significantly alters response to and recovery from brain injury, in part, *via* modulation of macrophage reactivity and cytokine production.

## Post-Injury Peripheral Immune Challenge Worsens Recovery Following TBI

Increasing evidence shows that TBI induces a persistent pro-inflammatory profile in microglia, but the functional consequence of this dysfunction is still under investigation. For example, single CCI in adult B6 mice induced chronic microglial reactivity 12 months post-injury. Highly reactive microglia were detected near the lesion cavity and characterized by increased expression of MHC-II, CD68, and NADPH oxidase (56). Midline FPI also induced features of primed pro-inflammatory microglia up to 1 week post-injury, which included elongated, rode-shaped Iba1^+^ cells that were also MHC-II and CD68 positive (203). Follow-up studies revealed that MHC-II mRNA and protein expression increased specifically in microglia after FPI and correlated with Iba1 reactivity and amoeboid morphology at 30 DPI (204). These data align with human studies showing a persistent post-injury microglial inflammatory profile. For example, inflammatory cytokines IL-6 and TNFα are detected in the CSF up to 12 months post-severe TBI and correlate with functional impairment and disinhibition (205, 206). Immunohistochemical analysis of autopsied brains revealed increased CD68^+^ and CR3/43^+^ (MHC-II^+^) reactive microglia several months post-injury. The presence of TAI increased the immunoload of microglial reactivity, particularly in the white matter (207). Separate studies demonstrate that CR3/43^+^ cells associate with increased APP accumulation 2 weeks post-injury and myelin basic protein 2–8 years post-injury (11). Together these data confirm that TBI induces persistent macrophage inflammation; however, the cell-specific role of microglia and monocytes remains unknown. Without cell-specific analysis of microglia and monocytes, the distinct role of each cell type is unclear and, therefore, not targetable with therapeutics. For example, macrophage reactivity could be the result of some other pathological process or macrophage reactivity could be perpetuating post-injury pathology. Additional work is needed to address these outstanding concerns.

Recent data demonstrate that TBI induces a primed microglial phenotype, defined by altered morphology and increased expression of MHC-II and CD68 (204, 208). Primed microglia do not display acute reactivity but instead become hyper-reactive after immune stimulation [see review in Ref. (66)]. This effect has been observed in experimental models of aging. For example, aged rodents display microglial priming via increased expression of MHC-II, complement receptor 3 (CD11b) and altered morphology (209, 210). Aged animals challenged with lipopolysaccharide (LPS) display increased microglial expression of IL-1β compared to adult mice, which results in prolonged sickness behavior (211) and a depressive-like phenotype (212). Follow-up studies revealed that LPS challenge in aged mice also promoted increased hippocampal expression of IL-1β, IL-6, and TNFα as well as spatial memory impairments (213, 214). Moreover, a similar effect is induced by midline FPI in adult mice. Midline FPI induces acute microglial activation, recruitment of peripheral cells, and motor impairment; however, many of these effects are transient. Only glial reactivity persists 30 DPI with deramified microglia maintaining increased expression of MHC-II. Peripheral LPS challenge at 30 DPI caused an exaggerated microglial response in TBI mice characterized by increased MHC-II, IL-1β, and TNFα expression and depressive-like behaviors compared to TBI mice given saline (204). Subsequent studies confirmed that LPS at 30 DPI exaggerated memory recall deficits in TBI mice as well (208). Another group has since reported that LPS 5 DPI causes an exaggerated inflammatory response in TBI rats (*via* impact acceleration) which is associated with depressive-like behavior and cognitive impairments 3 months post-injury (215). These results emphasize the chronic nature of the inflammatory response to TBI and confirm that subsequent post-injury immune challenges influence outcome and elicit exaggerated behavioral deficits.

Together, these studies indicate that primed microglia potentiate brain pathology and behavioral decline in aging and after CNS injury. While there are many similarities between age- and injury-related microglial priming, the cumulative effect of aging and TBI in microglial priming remains unknown and may be critical in determining the relationship between TBI and development of age-related neurodegenerative disease such as AD. For example, recent analysis of human brain samples revealed that LPS and *E. coli* K99 proteins were increased in AD brains compared to controls. LPS co-localized with Aβ40 and Aβ42 around amyloid plaques and near blood vessels (216). Multiple experimental studies show that peripheral LPS induced neuroinflammation, accumulation of Aβ, tau pathology, and cognitive impairment in non-transgenic rodents (217–219) although variable Aβ and tau pathology is apparent in transgenic mouse models of AD after peripheral LPS treatment (220–224). Moreover, peripheral LPS treatment after ischemia-hypoxia induced Aβ that co-localized with myelin aggregates in rats (225). While the mechanism(s) by which LPS enters the brain in unknown, these studies lend support to the notion that infection may be associated with development of AD. Thus, post-TBI infection that stimulates an inflammatory response may have a significant effect in long-term recovery.

### Repetitive TBI as a Post-Injury Immune Challenge

Mounting evidence indicates that neuroinflammation and microglial priming is a factor in repetitive TBI as well. In this case, the first TBI is the priming event and subsequent brain injuries cause an exaggerated inflammatory response that promotes pathology. Repetitive TBI through participation in contact sports is associated with chronic cognitive impairment, including development of CTE, a neurodegenerative disease characterized by abnormal tau accumulation in the sulci of the cortex (226). While Aβ plaques are present in some cases of CTE, the distribution and location is distinct from that occurring in AD (227–229). Glial reactivity is a common feature of CTE and includes astrocytic accumulation of abnormal tau and microglial reactivity (152, 226). For example, recent PET imaging studies reveal increased TSPO binding in retired NFL players in the hippocampus, entorhinal cortex, parahippocampal cortex, and supramarginal gyrus compared to age- and sex-matched controls without a history of repeated brain injury (230). This supports other studies showing increased CD68^+^ microglia in the brains of American football players, which partially mediated coincident tau pathology (231). Together, these data indicate that chronic neuroinflammation mediates AD-related pathology following repetitive TBI and the inter-injury time interval may be critical in this response.

One is faced with many challenges when trying to summarize data from experimental studies describing the relationship between repetitive TBI, neuroinflammation, and AD. First, a universal experimental model of repetitive TBI is not established. Thus, there is a great deal of variability in the number of injuries and timing between injuries in published reports. Second, the role of amyloid is underexplored in experimental models of repetitive TBI, which is predominantly characterized by tau pathology. Third, control animals after each TBI are not always included. For example, comparisons are often made between sham mice and brain injured animals that received the highest number of repetitive brain injuries. As a result, data describing inflammatory changes between injuries remain limited. Finally, most studies use Iba1 or morphological analysis to define reactive macrophages at a sub-acute time point after the final brain injury. Repetitive TBI consistently alters astrogliosis and microgliosis up to 1 year post-injury with a positive correlation between number of injuries and gliosis (232). While these data are important, they provide a restricted view of the cell-specific role of microglia and monocytes and do not define the cell-specific inflammatory state between injuries. To date, no studies could be found that define inter-injury macrophage changes in experimental models of repetitive TBI. Thus, the following paragraphs will briefly describe the incidence of Aβ and tau pathology as well as macrophage reactivity at post-injury time points following repetitive TBI.

Animal models of repetitive mild TBI are described in a recent review, which includes references to development of post-injury Aβ and tau pathology (232). While severity of repetitive TBI is typically referred to as “mild,” the number of injuries varies from 2 to 10 across several days or weeks. A recent highly repetitive mouse model of TBI including 30 injuries has also been characterized (59). Typically 1 or 2 brain injuries are administered per injury day. Non-transgenic mice exposed to repetitive mild TBI consistently show increased APP, phosphorylated tau, and behavioral impairment at chronic post-injury time points (232). Only two reports examining repetitive TBI in Tg2576 mice showed increased Aβ in addition to behavioral impairment at chronic post-injury time points (165, 233). Similarly, only transgenic tau mouse models [T44 (one mouse), hTau] displayed NFT pathology following repetitive TBI (153, 234). Macrophage reactivity was only reported in 1 of these 4 studies, and indicated that repetitive TBI in aged hTau mice resulted in increased CD45 immunoreactivity in the cortex, corpus callosum, and hippocampus 3 weeks post-injury (153). Together, these data are similar to single TBI experimental studies and show that TBI alone is not sufficient to induce amyloid or tau aggregation in non-transgenic rodents. The presence of pathological proteins (e.g., amyloid and tau oligomers or fibrils) in transgenic rodents at the time of TBI is sufficient to promote aggregation. Nonetheless, a recent report indicates that 30 mild TBI’s do not alter Aβ and tau pathology in 18-month-old 3xTg mice (59). In summary, these data confirm the complexity of repetitive TBI and strongly emphasize the need for more research in this area. Furthermore, a uniform experimental model is required to confirm the inter-relationship between repetitive TBI, neuroinflammation, and AD-like pathology.

What about peripheral immune challenge after repetitive TBI? One group considered this question and determined that timing of LPS treatment mediated a beneficial or detrimental post-injury effect in rats. For example, LPS treatment 1 day after repetitive mild TBI (3 TBI’s, 5 days apart) increased macrophage reactivity but decreased production of inflammatory cytokines and reduced neuronal injury (235). Delayed LPS treatment 5 days after repetitive TBI increased inflammatory cytokines, worsened neuronal damage including phosphorylation and aggregation of tau, and impaired behavioral recovery (235). These results highlight the temporal immune response to TBI and indicate that delayed post-injury immune challenges are detrimental to outcome.

## Macrophage Elimination Alters Recovery Following TBI

If the macrophage response is critical in mediating outcome following TBI, removal of microglia and/or monocytes should substantially alter recovery. Studies with CCR2 and CX3CR1 knock-in/knock-out mice demonstrate that permanent interruption of the microglial or macrophage response to TBI does not offer optimal protection after injury. Alternatively, various pharmacologic agents are available to transiently interrupt the microglial and macrophage response to injury. While the use of these agents is limited in experimental TBI studies, several groups have reported that microglial elimination [*via* colony-stimulating factor 1 receptor (CSF1R) inhibition] improves behavioral performance and synaptic functioning independent of Aβ accumulation in multiple transgenic mouse models of AD (3xTg, 5xFAD, APP/PS1) (236–238). Similarly, CSF1R inhibition after a chronic hippocampal lesion model of brain injury improved behavioral recovery, reduced pro-inflammatory molecules, and increased dendritic spines (239). Recent studies using the same injury model confirmed that post-injury microglial depletion followed by microglial repopulation improves behavioral recovery, attenuates the lesion-induced neuroinflammatory response, and increases dendritic spin densities despite extensive neuronal loss in the hippocampus (240). Another group examined the role of microglia in axonal damage following repetitive TBI by using CD11b-TK (thymidine kinase) mice, which require valganciclovir to deplete macrophages. In these experiments, low and moderate doses of valganciclovir reduced CD11b cell populations with no effect on axonal injury, silver staining, or APP accumulation at sub-acute post-injury time points (241). Discrepancies between the brain injuries studies are likely due to multiple factors, including (1) injury model; (2) post-injury time points; (3) post-injury outcome measures (only one considered the effect on APP and tau pathology); (4) CSF1R targets microglia specifically while CD11b^+^ cells include microglia and macrophages; and (5) CSF1R eliminated >90% of microglia while low and intermediate dose of valganciclovir depleted 35 and 56% of CD11b^+^ cells, respectively. Nonetheless, these studies highlight the potential therapeutic relevance of targeting microglia and macrophages to modulate post-injury outcome and further indicate that neuroinflammation is a critical mediator of post-injury pathology.

## Conclusion

**Figure 1:**
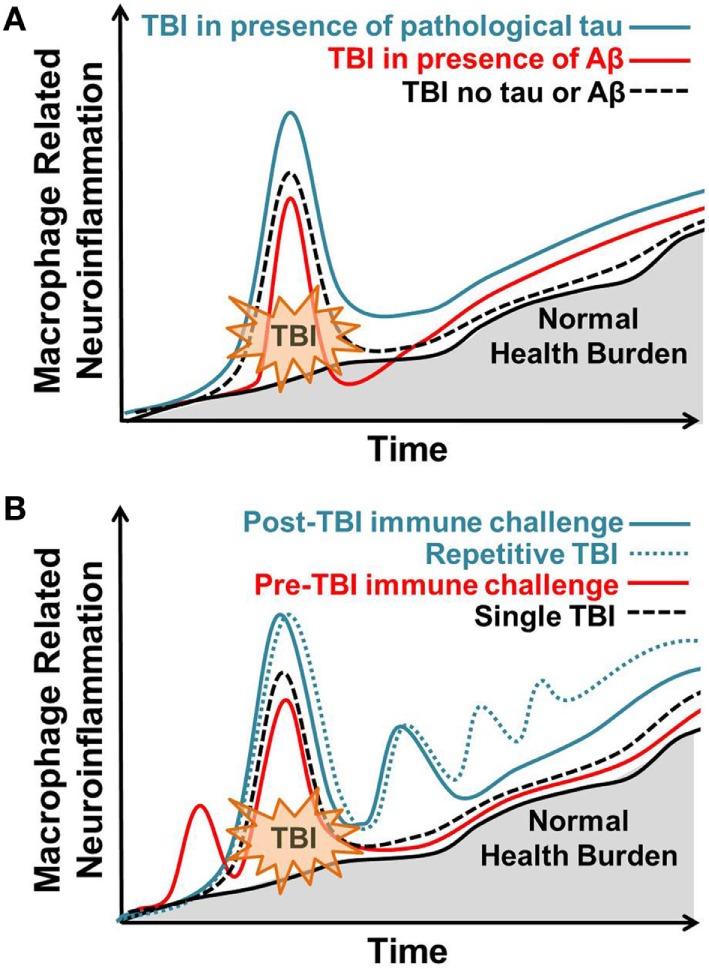
Macrophage-related response to brain injury varies in response to previous, coincident, and subsequent immune stressors. Normal, age-related health burden is depicted with a solid black line and gray shading. **(A)** Traumatic brain injury (TBI) in the presence of pathological tau (solid blue line) results in an enhanced macrophage response to TBI that remains elevated at chronic post-injury time points. TBI in the presence of Aβ (solid red line) results in an acute blunted macrophage response that increases at chronic post-injury time points. TBI occurring in the absence of tau or Aβ (dotted black line) results in acute macrophage-related neuroinflammation that subsides over time. **(B)** Post-injury peripheral immune challenge (solid blue line) causes a hyper-active macrophage response correlating with behavioral dysfunction. Repetitive post-injury immune challenge (dotted blue line), similar to what is observed in repetitive TBI, increases macrophage-related neuroinflammation and correlates with the advanced neuropathology. Pre-injury peripheral immune challenge at sub-threshold levels (red line) attenuates the post-injury macrophage-related inflammatory response to TBI. Single TBI (dotted black line) results in acute macrophage-related neuroinflammation that subsides over time. Over time, macrophage-related neuroinflammation increases with normal health burden.

Experimental models are a valuable resource in identifying the underlying biological pathways that link TBI to AD. Both TBI and AD are complex neurodegenerative pathologies that elicit a central and peripheral immune response. This review showcases the dynamic nature of post-injury macrophage-mediated inflammation in promoting post-injury Aβ and tau pathology. Throughout the review, several themes emerged that are notable and depicted in Figure 1. First, the inflammatory response to TBI is not absolute and is influenced by previous and subsequent inflammatory challenges. This is best reflected in studies with LPS administration before or after TBI. Importantly, in both instances LPS administration altered outcome from TBI. Certainly, there is a specific cascade of inflammatory events that occur after TBI, but these studies indicate that subtle alterations in this response are possible and can influence outcome. Second, accumulation of Aβ and tau phosphorylation are routinely considered primary dependent variables in experimental studies, but these pathological features do not often correlate with neuronal loss or behavioral impairment. Furthermore, many studies report that TBI does not influence Aβ and/or tau pathology leaving one to question the true role of these proteins in post-injury outcome. Both Aβ and tau phosphorylation are reported in normal aging and could, therefore, influence the brain’s response to TBI without causing AD. Thus, accumulation of Aβ and tau phosphorylation could be viewed as part of the injury process instead of a result of the injury. Third, additional factors must account for the resistance of rodents to develop Aβ and tau pathology after TBI. This could be due, in part, to intrinsic differences between human and rodent APP and tau. Given that multiple mouse models of AD display an altered inflammatory response to TBI, it is possible that accumulation of pathological proteins alters the neuroinflammatory environment in a way influences the brain’s response to injury. One could speculate and suggest that low-molecular weight pathological proteins “prime” the brain to respond to TBI. The role of Aβ and tau in this “priming” is potentially distinct and may include beneficial and detrimental consequences depending on age at injury and time of post-injury analysis. Finally, the distinct role of microglia and monocytes in TBI requires additional investigation and characterization. Targeting these cell types independently may provide new avenues for therapeutic intervention. Accumulating evidence shows that transient interruption of the macrophage response to TBI could improve outcome. Moving forward, we must appreciate the continuous nature of inflammation and consider previous, consequent, and subsequent immune challenges as mediators of post-injury outcome.

## Author Contributions

OK-C wrote the manuscript; JG provided expertise in the neuroinflammation and edited the manuscript.

## Conflict of Interest Statement

This research was conducted in the absence of any commercial or financial relationships that could be construed as a potential conflict of interest.
